# Denosumab and Calcium Homeostasis in Hypercalcaemia of Malignancy and Primary Hyperparathyroidism

**DOI:** 10.1155/ije/6930716

**Published:** 2026-11-10

**Authors:** Fengye Zhou, Ying Liu, Chunfang Liang, Sufang Yun

**Affiliations:** ^1^ Department of Pharmacy The Affiliated Hospital of Inner Mongolia Medical University Hohhot 010059 Inner Mongolia China nmgfy.com; ^2^ Graduate School Inner Mongolia Medical University Hohhot 010059 Inner Mongolia China immu.edu.cn; ^3^ Department of Endocrinology The Affiliated Hospital of Inner Mongolia Medical University Hohhot 010059 Inner Mongolia China nmgfy.com

**Keywords:** adverse effect, denosumab, hypercalcaemia, malignancy, off-label use, primary hyperparathyroidism

## Abstract

**Purpose:**

To evaluate short‐term changes in serum calcium and the occurrence of values below prespecified thresholds after denosumab treatment in institutional oncology and primary hyperparathyroidism (PHPT) cohorts, and to interpret these findings within the context of FAERS reporting patterns and published PHPT evidence.

**Methods:**

We retrospectively analysed denosumab‐treated patients at the Affiliated Hospital of Inner Mongolia Medical University; the retrospective study was performed in the Department of Clinical Pharmacy (oncology cohort, 2024; PHPT cohort, 2022–April 2025). Total serum calcium was evaluated at baseline and around Days 3 and 7. A downstream FAERS reanalysis used the fixed 2014Q1–2023Q2 dataset, and published PHPT reports were re‐extracted for actual follow‐up and complications.

**Results:**

In the oncology subgroup with baseline calcium > 2.55 mmol/L, mean paired calcium changed from 3.101 to 2.278 mmol/L at Day 3 (*n* = 12; *p* = 0.0002) and from 3.158 to 1.990 mmol/L at Day 7 (*n* = 11; *p* < 0.0001). In PHPT, the corresponding changes were 3.376 to 2.661 mmol/L at Day 3 (*n* = 9; *p* = 0.0038) and 3.351 to 2.289 mmol/L at Day 7 (*n* = 8; *p* = 0.0013). During Days 1–10, calcium < 2.10 mmol/L occurred in 57/102 oncology patients with non‐low baseline calcium and 4/9 PHPT patients; values < 1.60 mmol/L occurred in 3/102 and 0/9, respectively. Among 1433 FAERS hypocalcaemia reports, 1186 (82.76%) had at least one serious‐outcome code, most often hospitalisation/prolonged hospitalisation (769; 53.66%) or another medically important condition (725; 50.59%). Crude hypocalcaemia reporting proportions were higher for 120 mg than 60 mg, for male than female reports, and for tumour‐related than osteoporosis indications; the youngest evaluable age strata also had higher proportions, but denominators were small, and the comparisons were unadjusted.

**Conclusion:**

In the analysed cohorts, denosumab was followed by short‐term calcium reduction in patients with baseline hypercalcaemia and in the small PHPT cohort. Values below 2.10 mmol/L were common in early follow‐up, whereas values below 1.60 mmol/L were uncommon, and clinical hypocalcaemia was not systematically adjudicated. FAERS hypocalcaemia reports often carried serious‐outcome codes, mainly hospitalisation and other medically important conditions, without establishing incidence, causality or independent risk factors.

## 1. Introduction

Hypercalcaemia denotes total or ionised serum calcium above the assay‐specific upper reference limit; interpretation requires attention to albumin, pH and repeat testing when persistent disease is suspected. Acute severe or symptomatic values require immediate clinical assessment. Hypercalcaemia can lead to fatigue, nausea, constipation, confusion, cardiac arrhythmias, coma and death (DE) [1–3].

Primary hyperparathyroidism (PHPT) and hypercalcaemia of malignancy (HCM) are the most common causes of hypercalcaemia, accounting for approximately 90% of all cases [2]. PHPT is a common endocrine disorder with a prevalence of about 0.1%–1.0%, occurring predominantly in middle‐aged and older women [4, 5]. Approximately 84% of PHPT cases are caused by a single adenoma, with the remainder due to multigland hyperplasia or, rarely, parathyroid carcinoma [6]. HCM is a major complication of advanced cancer. About 2.7% of cancer patients develop HCM, and up to 30% of patients with advanced malignancies may experience hypercalcaemia during the disease course; it is widely regarded as a marker of poor prognosis [7, 8]. Mechanisms underlying HCM include tumour secretion of humoral factors leading to elevated parathyroid hormone (PTH)–related peptide, local osteolytic bone destruction with calcium release and coexistent PHPT [9].

The cornerstone of hypercalcaemia management is treatment of the underlying cause together with prompt calcium‐lowering therapy. Parathyroidectomy remains the definitive treatment for suitable patients with PHPT. For severe PHPT‐related hypercalcaemia before surgery, recent French guidance places rehydration at the foundation of care and recommends intravenous bisphosphonates when a sustained reduction is required; denosumab is a second‐line option when bisphosphonates cannot be used, particularly in renal impairment [10, 11].

Denosumab inhibits RANKL/RANK signalling and osteoclast‐mediated bone resorption. This can not only reduce bone‐derived calcium release but can also precipitate hypocalcaemia. A previous FDA Adverse Event Reporting System (FAERS) study identified a denosumab‐associated hypocalcaemia signal [12]. Denosumab has also been used for bisphosphonate‐refractory or contraindicated malignancy‐associated hypercalcaemia, with reports of marked calcium reduction [13].

The same antiresorptive mechanism underlies both the potential calcium‐lowering effect and the risk of oversuppression of calcium release [14]. We first evaluated real‐world data from denosumab‐treated cancer patients and patients with PHPT in our centre, placed these findings alongside FAERS pharmacovigilance data and integrated the existing literature to explore the potential off‐label use of denosumab in hypercalcaemia management, particularly in PHPT. Our aim was to provide evidence to support rational prescribing and risk management in clinical practice. The three sources were analysed separately rather than pooled, and FAERS reporting proportions were not interpreted as incidence or causal risk. To our knowledge, no previous study has combined large‐scale pharmacovigilance data with institutional cohorts and a review of published PHPT cases to characterise the potential use of denosumab in clearly defined hypercalcaemia scenarios.

## 2. Materials and Methods

### 2.1. Institutional Oncology Cohort

For the hospital‐based malignancy cohort, we retrospectively identified patients diagnosed with haematological malignancies or solid tumours who presented to our hospital between January 2024 and December 2024 and received denosumab treatment. This period was selected because a standardised institutional workflow for denosumab use and follow‐up in oncology‐related hypercalcaemia was implemented during this interval. For each patient, we collected age, sex, tumour type, denosumab dose and serum calcium levels before and after treatment. Patients were eligible if they were at least 18 years old; had essentially normal blood counts and liver and kidney function without contraindications to denosumab; and had complete clinical information, including medical history, laboratory tests and imaging findings. We excluded patients with uncontrolled active malignancy, known hypersensitivity to denosumab or its components, incomplete clinical data that precluded accurate assessment of efficacy and safety, coexisting disorders known to affect calcium metabolism such as osteomalacia or Paget’s disease, severe hypocalcaemia (serum calcium < 1.60 mmol/L) that might increase treatment risk and malabsorption syndromes or gastrointestinal diseases associated with impaired absorption.

### 2.2. Institutional PHPT Cohort

For the PHPT cohort, we retrospectively collected data for patients diagnosed with PHPT in the thyroid and breast surgery and endocrinology departments of our hospital who received denosumab between January 2022 and April 2025. The extended inclusion window reflected the low frequency of this off‐label indication and was intended to capture all consecutive eligible PHPT cases during the study period. Recorded variables included age, sex, denosumab dose, serum calcium before and after treatment, PTH, serum alkaline phosphatase (ALP), serum creatinine (Scr), baseline 25(OH)D when available, prior bisphosphonate or cinacalcet exposure and relevant complications. All PHPT patients were treated with a single 60‐mg subcutaneous dose of denosumab. We monitored changes in serum calcium, PTH and renal function indices during follow‐up.

### 2.3. Pharmacovigilance Analysis Using the FAERS Database

We obtained data from the publicly available US FAERS, spanning the first quarter of 2014 through the second quarter of 2023. Denosumab exposures were identified using drug names and active ingredient (‘denosumab,’ ‘Prolia’ and ‘Xgeva’); reports in which denosumab was coded as the primary suspect drug were retained and analysed at the deduplicated report (primaryid) level.

Calcium‐related events were defined using prespecified MedDRA preferred terms covering hypocalcaemia, hypercalcaemia and direction‐unspecified calcium abnormalities; the full preferred‐term list is provided in the Supporting Methods. Serious outcomes were defined by outcome codes for DE, life‐threatening (LT) events, hospitalisation (HO) or prolonged HO, disability (DS), congenital anomaly (CA), required intervention (RI) and other (OT) medically important conditions; a report could carry more than one code. Explicit 60 mg and 120 mg dose text, indication, age and sex were used for descriptive strata. Time to onset was calculated from the earliest valid denosumab start date to the event date and restricted to 0–3650 days.

Counts and crude reporting proportions with exact binomial 95% confidence intervals were reported. Fisher’s exact test described the unadjusted association between a calcium report and any coded serious outcome. Because FAERS lacks a treated‐population denominator and is subject to duplicate, missing, stimulated and selective reporting, the analysis does not estimate incidence, causality or independent risk factors.

### 2.4. Literature Search and Synthesis

To supplement our real‐world data, we conducted a computerised literature search from January 2012 to March 2025 in PubMed, Web of Science, SpringerLink, China National Knowledge Infrastructure (CNKI), the Wanfang Data knowledge platform and the VIP database. Search terms included ‘denosumab,’ ‘hypercalcemia,’ ‘hypercalcemia of malignancy,’ ‘primary hyperparathyroidism,’ ‘parathyroid carcinoma,’ ‘refractory hypercalcemia,’ ‘hypercalcemic crisis,’ ‘hypocalcemic drugs,’ ‘rebound hypercalcemia’ and ‘denosumab withdrawal.’ We included studies published in domestic or international journals that reported clearly diagnosed cases judged to be related to conditions treated with denosumab; provided detailed descriptions of denosumab administration and clinical response; and used designs such as randomised controlled trials, prospective or retrospective cohort studies, case series, systematic reviews, meta‐analyses or individual case reports with particular clinical relevance. Because most available publications were case reports or small cohorts with substantial heterogeneity in study objectives, follow‐up time points and concomitant treatment regimens, we restricted our synthesis to narrative and semiquantitative summaries of clinical characteristics and calcium‐lowering effects and did not perform a formal meta‐analysis.

### 2.5. Statistical Methods

Continuous variables are presented as mean ± standard deviation and categorical variables as counts and percentages. Pre‐ and posttreatment serum calcium values were compared using two‐sided paired *t*‐tests, including only patients with both baseline and follow‐up measurements; for the small PHPT samples, an exact paired sign‐flip test was added as a sensitivity analysis. No a priori sample‐size calculation was performed. Analyses were performed using *R* software.

## 3. Results

### 3.1. Institutional Oncology Cohort

Between 1 January and 31 December 2024, 172 cancer patients who received denosumab in our hospital were identified. Ninety‐six (55.81%) were men, and 76 (44.19%) were women, with a median age of 67 years (range: 36–79 years). Fifty‐eight patients (33.72%) had haematological malignancies, including 57 with multiple myeloma (33.14%) and 1 with chronic lymphocytic leukaemia (0.58%). The remaining 114 patients (66.28%) had solid tumours, including 60 with lung cancer (34.88%), 9 with breast cancer (5.23%), 7 with ureteral cancer, 5 with prostate cancer, 4 with colorectal cancer, 4 with cervical cancer and OT tumour types.

Baseline calcium was available in 171 patients: 69 were < 2.10 mmol/L, 87 were 2.10–2.55 mmol/L and 15 were > 2.55 mmol/L; one patient had no baseline or follow‐up calcium. Paired results are shown in Table 1. In the baseline‐hypercalcaemia group, mean calcium changed from 3.101 to 2.278 mmol/L at Day 3 (*n* = 12; mean change: −0.823 mmol/L, 95% CI: −1.150 to −0.497; *p* = 0.0002) and from 3.158 to 1.990 mmol/L at Day 7 (*n* = 11; mean change: −1.168 mmol/L, 95% CI: −1.507 to −0.829; *p* < 0.0001). These within‐group changes are vulnerable to regression to the mean and co‐intervention confounding.

**TABLE 1:** Paired serum calcium values in the denosumab‐treated oncology cohort.

Target	Baseline group	Paired *n*	Paired baseline	Follow‐up Ca	Mean change (95% CI); *p*
Day 3	< 2.10	35	1.975	1.937	−0.038 (−0.094 to 0.018); 0.1755
Day 3	2.10–2.55	36	2.285	2.048	−0.237 (−0.297 to −0.177); < 0.0001
Day 3	> 2.55	12	3.101	2.278	−0.823 (−1.150 to −0.497); 0.0002
Day 7	< 2.10	51	1.955	1.941	−0.014 (−0.061 to 0.034); 0.5636
Day 7	2.10–2.55	63	2.255	2.026	−0.229 (−0.279 to −0.179); < 0.0001
Day 7	> 2.55	11	3.158	1.990	−1.168 (−1.507 to −0.829); < 0.0001

Among patients with baseline calcium ≥ 2.10 mmol/L and at least one Days 1–10 follow‐up value, 57/102 (55.9%) had calcium < 2.10 mmol/L and 3/102 (2.9%) had < 1.60 mmol/L. The corresponding counts were 49/87 and 1/87 in the baseline 2.10–2.55 mmol/L group, and 8/15 and 2/15 in the baseline > 2.55 mmol/L group. Among the 69 patients with baseline calcium < 2.10 mmol/L, 61 had a subsequent value of < 2.10 mmol/L and one had < 1.60 mmol/L. Symptoms, event‐directed treatment and causality were not systematically recorded.

### 3.2. Institutional PHPT Cohort

The PHPT cohort comprised 10 patients (three men and seven women), with a median age of 72 years (range: 31–87). Individual patient characteristics, denosumab dosing, concomitant interventions and longitudinal laboratory measurements are presented in Supporting Table 1. Nine baseline/Day‐3 pairs had mean calcium of 3.376 ± 0.489 and 2.661 ± 0.341 mmol/L (mean change: −0.715 mmol/L, 95% CI: −1.124 to −0.306; *p* = 0.0038). Eight baseline/Day‐7 pairs had means of 3.351 ± 0.517 and 2.289 ± 0.289 mmol/L (mean change: −1.063 mmol/L, 95% CI: −1.551 to −0.574; *p* = 0.0013) (Table 2); exact sign‐flip sensitivity *p* values were 0.0078 at both time points. Four of nine patients with baseline calcium ≥ 2.10 mmol/L had a Days 1–10 laboratory value of < 2.10 mmol/L, and none had < 1.60 mmol/L. One threshold crossing followed surgery on Day 2; after censoring from surgery, 3/8 evaluable patients crossed the threshold.

**TABLE 2:** Paired serum calcium values in the institutional PHPT cohort.

Comparison	Paired n	Baseline mean ± SD	Follow‐up mean ± SD	Mean change (95% CI)	*p*
Baseline to Day 3	9	3.376 ± 0.489	2.661 ± 0.341	−0.715 (−1.124 to −0.306)	0.0038
Baseline to Day 7	8	3.351 ± 0.517	2.289 ± 0.289	−1.063 (−1.551 to −0.574)	0.0013

Symptoms, event‐directed treatment and attribution were not systematically documented. Given incomplete sampling, heterogeneous follow‐up timing and incompletely documented units, the remaining biochemical measures are reported descriptively only, with no inferential comparisons (values are given as baseline and follow‐up, respectively): PTH 506.4 ± 424.3 and 398.5 ± 562.4 pg/mL (*n* = 8); ALP 196.5 ± 137.9 and 181.5 ± 133.6 U/L (*n* = 2); Scr 212.8 ± 111.5 and 184.8 ± 90.6 μmol/L (*n* = 5); eGFR 100.7 ± 36.2 and 73.7 ± 11.0 mL/min/1.73 m^2^ (*n* = 3); and baseline 25(OH)D median 14.00 ng/mL (IQR: 11.81–16.86, range: 9.94–18.80; *n* = 8).

### 3.3. Published PHPT Cases Treated With Denosumab

Full‐text re‐extraction identified 18 publications containing 160 reported PHPT participant entries [15–32]. Five cohorts or trials contributed 144 entries, and 13 case reports or small series contributed 16 entries. This total counted 30 evaluable participants from the DENOCINA trial, to which 31 participants had originally been assigned and of whom one withdrew. Two Russian cohorts arose from the same centre during overlapping periods, so unique‐patient overlap cannot be excluded. A definitive morphologic or pathologic subtype was reported in 20 patients, including 9 with parathyroid carcinoma, 9 with typical parathyroid adenoma and 2 with atypical adenoma; the remaining entries had clinically or biochemically diagnosed PHPT without a consistently documented subtype. Because the included studies differed substantially in treatment indication, calcium measurement, follow‐up duration and concomitant therapy, overall pooled baseline calcium and PTH values were not calculated. In reports of patients with baseline hypercalcaemia, 14 patients had traceable paired calcium measurements obtained on Days 2–4, with mean serum calcium decreasing from 3.72 ± 0.36 to 3.06 ± 0.38 mmol/L (mean reduction: 0.66 mmol/L), and 15 patients had paired measurements obtained on Days 5–9, with mean calcium decreasing from 3.76 ± 0.50 to 2.67 ± 0.20 mmol/L (mean reduction: 1.09 mmol/L). These findings were summarised descriptively because many patients received concomitant treatments, and observations were clustered within individual studies; no inferential *p* values were calculated. Follow‐up was within 1 month in four publications, up to 12 months in seven and beyond 12 months in seven. Hypocalcaemia was described in seven publications: six DENOCINA participants receiving denosumab plus cinacalcet; one asymptomatic Grade‐2 biochemical event in the 19‐patient Miyaoka cohort; and five publications in severe‐PHPT settings with perioperative or hungry‐bone‐context hypocalcaemia, including one case requiring calcium and calcitriol for more than three years. Rebound hypercalcaemia after withdrawal was described in one case. Detailed study‐level information is provided in Supporting Table 2.

### 3.4. FAERS Report‐Level Findings

The fixed dataset contained 97,348 reports during 2014Q1–2023Q2, of which 75,493 included denosumab as the primary suspect drug. Core calcium‐homeostasis PTs were present in 1885 reports (2.50%, 95% CI: 2.39%–2.61%): 1433 contained a hypocalcaemia PT, 419 a hypercalcaemia PT and 39 both. Any coded serious outcome was present in 1513/1885 (80.27%) core‐calcium reports and 38,308/73,608 (52.04%) reports without a core‐calcium PT (Fisher OR: 3.748, 95% CI: 3.341–4.213; *p* < 0.001). Among hypocalcaemia reports, 1186/1433 (82.76%) had a serious‐outcome code. HO/prolonged HO occurred in 769/1433 (53.66%), OT medically important conditions in 725/1433 (50.59%), LT events in 122/1433 (8.51%), DEs in 111/1433 (7.75%), DS in 29/1433 (2.02%), RI in 11/1433 (0.77%) and CA in 2/1433 (0.14%). Codes were not mutually exclusive, and hypocalcaemia was not established as the cause of the outcome.

The crude hypocalcaemia reporting proportion was 4.73% (290/6125) for explicit 120 mg reports and 1.69% (478/28,281) for explicit 60 mg reports; 41,087 reports had OT or unknown dose information. It was 4.58% (471/10,288) in male and 1.50% (938/62,680) in female reports, with 2525 reports of unknown/OT sex. By indication, proportions were 6.10% (357/5853) for tumour/neoplasm‐related, 1.89% (634/33,614) for osteoporosis‐related and 1.22% (428/35,145) for OT classified indications; 881 reports lacked a classified indication. By age, proportions were 7.62% (8/105) at 18–24 years, 4.07% (27/663) at 25–39, 3.53% (50/1418) at 40–49, 1.97% (309/15,657) at 50–64, 1.83% (662/36,089) at 65%–79% and 1.75% (377/21,561) at ≥ 80. These unadjusted differences may reflect indication, disease severity, exposure, monitoring and reporting bias; small younger‐age denominators require particular caution.

Valid onset timing was available for 473/1433 hypocalcaemia reports. The median was 14 days (IQR: 6–64), and 306/473 (64.7%) occurred within 30 days. EVENT_DT was report‐level rather than PT‐specific, so timing was descriptive.

## 4. Discussion

This study integrated FAERS pharmacovigilance data, real‐world clinical evidence from malignancy‐associated hypercalcaemia and PHPT patients in our centre and published case reports to comprehensively evaluate the regulatory effects of denosumab on calcium metabolism and their clinical implications. The largest paired calcium reductions occurred in oncology patients with baseline hypercalcaemia and in the small PHPT cohort, whereas values in patients with normal or low baseline calcium changed little and generally remained within or near the reference range. This baseline dependence is expected for an antiresorptive agent: when bone resorption drives hypercalcaemia, its inhibition can correct calcium substantially, but the same mechanism offers little additional lowering when calcium is already normal. It also implies that the risk of clinically important hypocalcaemia depends more on baseline calcium and patient characteristics than on dose alone. Baseline stratification, irregular sampling, surgery and OT calcium‐lowering treatment can produce regression to the mean and confounding; the changes are therefore not comparative treatment effects. More than half of evaluable oncology patients with a nonlow baseline had an early value of < 2.10 mmol/L, but the source tables did not systematically capture symptoms, treatment or attribution. We therefore describe laboratory threshold crossings rather than adjudicated hypocalcaemia adverse reactions.

Published reports support a possible short‐term role for denosumab when conventional treatment is ineffective or unsuitable, but the evidence is dominated by small, heterogeneous studies with frequent co‐interventions. Denosumab should therefore be considered an adjunct in selected cases rather than a substitute for definitive parathyroid surgery or established acute therapy.

FAERS made the serious‐outcome wording more specific: HO and OT medically important conditions were the most frequent codes among hypocalcaemia reports, while LT events and DEs were less frequent. An independent FAERS analysis reported the same pattern, with a clear hypocalcaemia signal for denosumab and mostly reversible events after drug withdrawal and supportive treatment [12]. The crude differences across age, sex, dose and indication are signals for monitoring and hypothesis generation, not independent risk factors or grounds for dose selection.

In China, denosumab is approved only for osteoporosis and cancer‐related skeletal events, and its use in hypercalcaemia, particularly in nonmalignant conditions such as PHPT, remains off‐label. Parathyroidectomy is the only curative treatment for PHPT, and calcimimetics such as cinacalcet are the on‐label medical option for chronic or mild‐to‐moderate hypercalcaemia in patients who are not surgical candidates. Within this framework, published reports have used denosumab for severe or refractory PHPT‐related hypercalcaemia, mainly when rapid preoperative calcium control is required, or surgery is temporarily unsuitable, typically as a single 60‐mg dose with repeat dosing in some cases [15, 16]. The present results should be read within this regulatory and therapeutic context.

For PHPT, recent French guidance emphasises rehydration and intravenous bisphosphonates when sustained preoperative calcium lowering is required; denosumab is a second‐line option when bisphosphonates cannot be used [10, 11]. Consistent with this supportive role, PTH in our cohort remained highly variable without a consistent directional pattern, supporting the interpretation that denosumab provides symptomatic calcium control rather than mechanistic endocrine therapy and that any PTH changes are compensatory [16]. The literature includes single 60‐mg bridging doses and repeated 60–120 mg regimens, but postoperative hungry‐bone syndrome and denosumab exposure are often inseparable. Published PHPT evidence also shows why follow‐up should extend beyond the first week when treatment continues, surgery follows or denosumab is withdrawn. Prolonged hypocalcaemia and rebound hypercalcaemia have been described, and rebound timing may relate to cumulative exposure in a different paediatric indication [24, 30, 33]. These observations provide context but cannot be attributed to the institutional cohorts.

Limitations include the single‐centre retrospective design, irregular calcium sampling, incomplete documentation of dose, vitamin D and co‐treatment and no systematic adverse‐event collection. One oncology patient lacked baseline and follow‐up calcium, and because names and hospital numbers were masked and scrambled, treating 172 source rows as 172 different patients rests on investigator confirmation and cannot be independently revalidated. PHPT numbers were too small for multivariable modelling. The FAERS component used a prefiltered derived dataset whose upstream extraction cannot be reconstructed, and spontaneous reports are subject to missing, duplicate and selective reporting. Published reports are small, heterogeneous and may contain overlapping participants and publication bias.

Denosumab was followed by short‐term calcium reduction in patients with baseline hypercalcaemia and in the small PHPT cohort. Values below 2.10 mmol/L were common in early follow‐up, but clinical hypocalcaemia was not systematically adjudicated. FAERS hypocalcaemia reports often carried serious‐outcome codes, mainly HO and OT medically important conditions, without establishing incidence, causality or independent risk factors.

## Author Contributions

Fengye Zhou: data curation and formal analysis. Ying Liu: visualization. Sufang Yun: data curation and visualization. Chunfang Liang: conceptualization, methodology and supervision.

## Funding

This work was supported by the Natural Science Foundation of Inner Mongolia Autonomous Region (2023QN08012), the Joint Project of Inner Mongolia Medical University (YKD2023LH062), the Science and Technology Project of Public Hospital Research Joint Fund (2024GLLH0329) and the Specialized Regional Medical Centre of Inner Mongolia Autonomous Region.

## Ethics Statement

All procedures involving human participants were conducted in accordance with the Declaration of Helsinki. The pharmacovigilance component used publicly available FAERS data with no identifiable personal information; therefore, separate IRB approval and informed consent were not required for that component. The real‐world hospital component was approved by the Ethics Committee of the Affiliated Hospital of Inner Mongolia Medical University (Approval No.S.2026168). Written informed consent was waived by the Ethics Committee because of the retrospective design and use of de‐identified data.

## Consent

The authors have nothing to report. Please see the Ethics Statement.

## Conflicts of Interest

The authors declare no conflicts of interest.

## Supporting Information

Additional supporting information can be found online in the Supporting Information section.

## Supporting information


**Supporting Information** The supporting information contains additional methodological details for the FAERS downstream audit, available patient‐level clinical and biochemical data for the institutional PHPT cohort (Supporting Table 1) and publication‐level characteristics and treatment information for the 18 studies included in the PHPT literature review (Supporting Table 2).

## Data Availability

The data that support the findings of this study are openly available in the FDA Adverse Event Reporting System (FAERS) at https://fis.fda.gov/extensions/FPD-QDE-FAERS/FPD-QDE-FAERS.html. These data were derived from the following resources available in the public domain: FDA FAERS quarterly data extract files.
