# Supplementary material for: Denosumab and Calcium Homeostasis in Hypercalcaemia of Malignancy and Primary Hyperparathyroidism
Source: Int J Endocrinol. 2026 Nov 10;2026:6930716. doi: 10.1155/ije/6930716 (PMC13749068; doi:10.1155/ije/6930716)
Supplement: Supplementary file 1 [file IJE-2026-6930716-s001.docx]

**Supplementary Materials**

The supplementary material contains additional methodological details for the FAERS downstream audit, available patient-level clinical and biochemical data for the institutional PHPT cohort (Supplementary Table 1), and publication-level characteristics and treatment information for the 18 studies included in the PHPT literature review (Supplementary Table 2).

Supplementary Table 1. Available clinical and biochemical parameters of the institutional PHPT cohort

| **Case** | **Sex** | **Age band** | **Baseline Ca** | **Day-3 Ca** | **Day-7 Ca** | **Day-9 Ca** | **Early surgery** |
| --- | --- | --- | --- | --- | --- | --- | --- |
| PHPT01 | M | 65–79 | NR | 2.58 | 2.74 | NR | NR |
| PHPT02 | F | 65–79 | 3.57 | 2.58 | NR | NR | NR |
| PHPT03 | F | ≥80 | 3.08 | 3.11 | 2.49 | 2.18 | NR |
| PHPT04 | M | 40–49 | 3.86 | 2.41 | 2.08 | 2.08 | NR |
| PHPT05 | F | ≥80 | 4.11 | 2.88 | 2.30 | 2.11 | NR |
| PHPT06 | M | 50–64 | 2.63 | 2.56 | 2.04 | 1.85 | NR |
| PHPT07 | F | 50–64 | 2.78 | 2.07 | 2.11 | NR | Day 2 |
| PHPT08 | F | ≥80 | 3.22 | 2.82 | 2.70 | 2.57 | NR |
| PHPT09 | F | 25–39 | 3.50 | 3.08 | 2.64 | 2.54 | Day 12 |
| PHPT10 | F | 65–79 | 3.63 | 2.44 | 1.95 | 1.93 | NR |

Denosumab exposures were identified in the DRUG and DRUG_ALL tables by drug name (DRUGNAME) and active ingredient (prod_ai) using the keywords 'denosumab', 'Prolia' and 'Xgeva'. Reports with denosumab coded as primary suspect (ROLE_COD = 'PS') were retained, deduplicated by primaryid, and linked to the DEMO table for demographic characteristics and event dates.

The prespecified preferred-term list covered hypocalcaemia (e.g. HYPOCALCAEMIA, BLOOD CALCIUM DECREASED, CALCIUM IONISED DECREASED), hypercalcaemia (e.g. HYPERCALCAEMIA, BLOOD CALCIUM INCREASED, CALCIUM IONISED INCREASED) and direction-unspecified abnormalities (e.g. BLOOD CALCIUM ABNORMAL, CALCIUM IONISED ABNORMAL, CALCIUM METABOLISM DISORDER); calcification-related terms were not combined with this core outcome. A report with at least one listed preferred term in the REAC table was classified as a calcium-related report.

Dose records were normalised from DOSE_VBM text and brand names: records containing '60' or corresponding to the standard osteoporosis regimen were assigned to the 60 mg group, those containing '120' or corresponding to the high-dose oncologic regimen to the 120 mg group, and all others to other/unknown. Indications (INDI_PT) were classified as tumour/neoplasm-related, osteoporosis-related or other using disease keywords, with a hierarchical priority of malignancy, then osteoporosis, then other. Age was grouped as <18, 18–24, 25–39, 40–49, 50–64, 65–79 and ≥80 years. Time to onset required complete YYYYMMDD start and event dates and was restricted to 0–3650 days.

Supplementary Table 2. Published PHPT reports included in the full-text re-extraction

| **Study** | **n** | **Denosumab/co-intervention** | **Actual follow-up** | **Hypocalcaemia/treatment** | **Other outcome/interpretation** |
| --- | --- | --- | --- | --- | --- |
| Eller-Vainicher 2018 ^15^ | 25 | 60 mg every 6 months | 24 months | NR | Fractures reported; calcium AE not systematic |
| Eremkina 2020 ^16^ | 10 | Single 60 mg; saline/cinacalcet common | Days 3, 7, 9; surgery/1 month | Six postoperative cases; oral calcium/vitamin D | Hungry-bone/surgery confounding |
| Gronskaya 2023 ^17^ | 60 | 60 mg every 6 months; vitamin D | 24 months | No severe event in PHPT groups | One mild event in control group |
| Leere 2020 ^18^ | 30 | 60 mg baseline and 6 months; 14 also cinacalcet | Every 4 weeks to 52 weeks | 6/14; two received calcium | Six non-fatal serious AEs; none treatment-related |
| Calapkulu 2020 ^19^ | 1 | 120 mg monthly plus cinacalcet | 6 months | NR | No specific denosumab AE reported |
| Gill 2023 ^20^ | 1 | Dose NR; multiple acute co-treatments | 5 days to surgery; 4 weeks to death | No low calcium described | Death after anticoagulation complication; not attributed to denosumab |
| Jumpertz VSR 2015 ^21^ | 1 | 60 mg repeated; six doses | >2 years | None reported | No clinically relevant side effect reported |
| Kuchay 2021 ^22^ | 4 | Single 60 mg | 14 days; surgery at 2–8 weeks | Two postoperative cases; calcium/vitamin D | Hungry-bone/surgery context |
| Lenherr-Taube 2020 ^23^ | 1 | Single 60 mg after pamidronate | Day 8, surgery, 18 months | Prolonged postoperative; IV then oral calcium/calcitriol | Denosumab contribution not excluded |
| Li 2020 ^24^ | 1 | Single 120 mg before tumour debulking | >4 years | Severe, prolonged; calcium/calcitriol >3 years | After 120 mg and tumour debulking |
| Makino 2022 ^25^ | 1 | 120 mg with evocalcet/zoledronate/sorafenib | About 1 year; redosed | NR | Multiple co-treatments |
| Rajan 2019 ^26^ | 1 | Single 60 mg | Surgery 5 days later | No hungry-bone syndrome reported | No hypocalcaemia described |
| Roukain 2021 ^27^ | 1 | Nine 60/120 mg doses over 20 months | 3 years | NR | No hypocalcaemia reported; hypophosphataemia discussed |
| Shalaby 2014 ^28^ | 1 | Single 60 mg | 9 months | NR | No specific complication reported |
| Tzotzas 2022 ^29^ | 1 | 60 mg then 120 mg monthly plus cinacalcet | 36 months | None reported | No significant side effect; breakthrough hypercalcaemia |
| Camponovo 2020 ^30^ | 1 | 60 mg every 6 months; stopped then restarted | Rebound about 9 months after last dose | No hypocalcaemia reported | Severe symptomatic rebound; hospitalised |
| Miyaoka 2020 ^31^ | 19 | 60 mg every 6 months | Day 7, 6 and 12 months | 1/19 grade-2 biochemical event; asymptomatic | Retrospective comparison |
| Vellanki 2014 ^32^ | 1 | 120 mg then 60 mg as needed | Approximately 12 months | Postoperative nadir 6.9 mg/dL | Denosumab plus hungry-bone considered |

Ca: total serum calcium (mmol/L); NR: not reported or not recorded; PHPT: primary hyperparathyroidism. A blank/NR entry was not interpreted as absence of an even
